# Appropriateness of Bolus Antihypertensive Therapy for Elevated Blood Pressure in the Emergency Department

**DOI:** 10.5811/westjem.2017.5.33410

**Published:** 2017-07-11

**Authors:** Joseph B. Miller, Andrew Arter, Suprat S. Wilson, Alexander T. Janke, Aaron Brody, Brian P. Reed, Phillip D. Levy

**Affiliations:** * Henry Ford Hospital, Department of Emergency Medicine, Detroit, Michigan; † Detroit Medical Center, Detroit Receiving Hospital, Detroit, Michigan; ‡ Yale School of Medicine, Department of Emergency Medicine, New Haven, Connecticut; § Wayne State University, Department of Emergency Medicine, Detroit, Michigan; ¶ Wayne State University, Cardiovascular Research Institute, Detroit, Michigan

## Abstract

**Introduction:**

While moderate to severely elevated blood pressure (BP) is present in nearly half of all emergency department (ED) patients, the incidence of true hypertensive emergencies in ED patients is low. Administration of bolus intravenous (IV) antihypertensive treatment to lower BP in patients without a true hypertensive emergency is a wasteful practice that is discouraged by hypertension experts; however, anecdotal evidence suggests this occurs with relatively high frequency. Accordingly, we sought to assess the frequency of inappropriate IV antihypertensive treatment in ED patients with elevated BP absent a hypertensive emergency.

**Methods:**

We performed a retrospective cohort study from a single, urban, teaching hospital. Using pharmacy records, we identified patients age 18–89 who received IV antihypertensive treatment in the ED. We defined treatment as inappropriate if documented suspicion for an indicated cardiovascular condition or acute end-organ injury was lacking. Data abstraction included adverse events and 30-day readmission rates, and analysis was primarily descriptive.

**Results:**

We included a total of 357 patients over an 18-month period. The mean age was 55; 51% were male and 93% black, and 127 (36.4%) were considered inappropriately treated. Overall, labetalol (61%) was the most commonly used medication, followed by enalaprilat (18%), hydralazine (18%), and metoprolol (3%). There were no significant differences between appropriate and inappropriate BP treatment groups in terms of clinical characteristics or adverse events. Hypotension or bradycardia occurred in three (2%) patients in the inappropriate treatment cohort and in two (1%) patients in the appropriately treated cohort. Survival to discharge and 30-day ED revisit rates were equivalent.

**Conclusion:**

More than one in three patients who were given IV bolus antihypertensive treatment in the ED received such therapy inappropriately by our definition, suggesting that significant resources could perhaps be saved through education of providers and development of clearly defined BP treatment protocols.

## INTRODUCTION

Over half of patients with chronic hypertension have uncontrolled blood pressure (BP),^1^ a problem particularly prevalent in urban, African-American communities.^2^–^4^ Hypertension is frequently encountered in the emergency department (ED),^5^ and differs management widely.^6^ Despite the existence of evidence-based clinical policy statements by the American College of Emergency Physicians (ACEP) discouraging acute BP reduction in hypertensive patients who lack acute end-organ injury,^7^ and studies suggesting no harm without treatment,^8^ emergency physicians often feel compelled to administer antihypertensive therapy when systolic BP is markedly elevated.^9^ Even so, the incidence of true hypertensive emergencies among ED patients with or without chronic hypertension is well below 1%, and post-discharge adverse events are uncommon,^10^,^11^ suggesting that such concerns are largely unfounded.^12^

Inappropriate administration of antihypertensive therapy to patients without a hypertensive emergency, especially bolused intravenous (IV) medication, is not without risk and represents avoidable resource utilization.^13^ While evidence suggests that inappropriate IV antihypertensive therapy occurs with relative frequency in the ED,^8^,^11^ no prior study has sought to specifically characterize the appropriateness of bolus IV antihypertensive administration in ED patients with elevated BP.

## METHODS

### Study Design and Setting

We performed a retrospective cohort study of patients who presented to the ED of an urban, teaching hospital from January 2011 to July 2012. The hospital had a total annual census of approximately 110,000 adult patients during the study period, more than 80% of whom are African-American. Our institutional review board approved the study prior to data abstraction.

### Selection of Participants

Patients aged 18 to 89 years who received one or more IV bolus doses of labetalol, hydralazine, enalaprilat, or metoprolol in the ED were identified by a query of electronic pharmacy orders. We selected these four bolus-dosed medications as they are most often used to manage elevated BP in the ED and, unlike antihypertensive infusions, are more likely (though not exclusively) to be used in patients for whom there is uncertainty about a true hypertensive emergency.^6^ Use of pharmacy orders rather than baseline BP to identify the study cohort was deliberate, allowing us to efficiently address our study aim, which was to evaluate appropriateness of bolus IV antihypertensive therapy, and not the ED management of hypertension itself.

Once patients were identified, a single investigator performed chart abstraction using a predefined data dictionary and compiled demographic, medical history and clinical information for each patient including presenting symptoms, ED vital signs and ED laboratory data. Potential adverse effects related to antihypertensive treatment during the ED or hospital stay were also tracked. These adverse effects included documented hypotension, bradycardia (heart rate < 55 beats per minute), and syncope. In addition, abstraction included in-hospital mortality and ED repeat visits within 30 days. A second, independent investigator performed double chart abstraction on a random selection of 40 cases. Data were cross-checked for internal consistency and showed high agreement (> 95%). All data were obtained from the health system electronic medical record (EMR).

Population Health Research Capsule

What do we already know about this issue?

Severely elevated blood pressure is common in emergency care. Hypertensive emergencies, however, are rare.

What was the research question?

We hypothesized that bolus intravenous antihypertensive treatment occurs frequently when hypertensive emergencies are neither suspected nor present.

What was the major finding of the study?

We found that one in three patients inappropriately received bolus antihypertensive treatment.

How does this improve population health?

Avoidance of such treatment has the potential to reduce cost and reduce potential complications across populations with severe blood pressure elevation.

### Methods and Measurements

To determine whether or not the use of the IV antihypertensive bolus was appropriate, investigators pre-defined that the following four scenarios qualified as appropriate use. First, treatment was appropriate if administered to a patient with a documented hypertensive emergency, inclusive of acute myocardial infarction, acute heart failure or cardiogenic pulmonary edema, acute aortic dissection, acute stroke (hemorrhagic or ischemic), acute subarachnoid hemorrhage, hypertensive encephalopathy, preeclampsia/eclampsia, or acute renal failure. Second, treatment was appropriate if administered to a patient in whom there was ED documentation expressing concern for a potential hypertensive emergency but this diagnosis was not confirmed. Third, treatment was appropriate if administered to a patient in whom documentation indicated that a reason for hospital admission was further workup of a possible hypertensive emergency. Fourth, treatment was appropriate if administered to a patient with documented inadequate response to oral medications. Investigators considered treatment inappropriate if the patient received treatment solely for chronic, uncontrolled hypertension, the patient had no specified workup for hypertension (such as electrocardiogram or serial cardiac biomarkers), the patient was discharged from the ED or admitted to the hospital without any diagnoses related to hypertension, or the patient was admitted with a diagnosis of hypertension diagnosis without associated symptoms or clinical findings of end-organ damage.

### Statistical Analysis

Statistical analysis was primarily descriptive. We present mean values with associated standard deviation (SD). Group comparisons were performed using t-tests and chi-square or Fisher exact test as appropriate. A p-value of < 0.05 was considered statistically significant. We conducted all data analysis using SAS 9.0 (Cary, NC).

## RESULTS

### Characteristics of Study Participants

Over the study period, we identified 411 patients who received bolus IV antihypertensive medications, 54 (13.1%) of whom were excluded, primarily for age and antihypertensive administration, after the ED visit (Figure). Baseline characteristics for the final sample of 357 are shown in Table 1. Mean (SD) age was 54.7 (14) years, and patients were mostly African American (93%) with a high prevalence of known underlying chronic hypertension (88.2%). The mean (SD) initial ED BP for all patients was 201/114 (30/22) mm Hg. The mean (SD) BP post-treatment at 30 minutes was 177/100 (29/20) mmHg (n=217), difference −24/14 mmHg (12% SBP reduction). The mean (SD) BP post-treatment at 60 minutes was 176/97 (27/19) mmHg (n=207), difference from baseline −25/17 mmHg (12% SBP reduction).

Table 2 shows the antihypertensive agents administered to patients. Overall, 91% of patients received a single IV antihypertensive dose. Labetalol was the most common medication administered (60.8%), followed by enalaprilat (18.2%), hydralazine (17.9%), and metoprolol (3.1%).

### Main Results

As shown in Figure, 230 out of 357 patients received antihypertensive treatment for suspected or confirmed hypertensive emergency (64.4%) and met criteria for appropriate treatment. The majority of these patients had a primary ED diagnosis of hypertensive emergency (n=88; 38.3%), were evaluated in the ED for hypertensive emergency with an alternate primary diagnosis (n=78; 33.9%), or were admitted to the hospital for further workup of hypertensive emergency (n=54; 23.5%). In the inappropriate treatment group, the most common diagnosis was uncontrolled hypertension (n=52; 40.9%). There were 37 (29.1%) patients in this group who were discharged from the ED with no hypertension-related workup or diagnosis, and 12 (9%) patients were admitted to the hospital without a hypertension-related diagnosis. Compared to the appropriate treatment group, these patients were younger and less likely to have a prior history of cardiovascular disease. The patients in the inappropriate treatment group were also less likely to present to the ED with dyspnea, chest pain or confusion.

Patients were markedly hypertensive in both groups with no difference between in average initial BP. Baseline BP (SD) was 202/115 (29/23) mmHg in the appropriate treatment group and 198/112 (31/19) mmHg in the inappropriate treatment group (p=0.23). A majority of patients in the appropriate group (n=210, 91%) and in the inappropriate group (n=115, 91%) received a single bolus of medication (p=0.81). As show in Table 2, labetalol was used with similar frequency while enalaprilat administration was more commonly administered in the appropriate treatment group (22% vs 11%, p< 0.01).

There was no difference in mean (SD) BP post-treatment at 30 or 60 minutes between groups. Blood pressure in the appropriate group was 178/100 (31/21) mmHg compared to 176/98 (25/17) mmHg in the inappropriate group at 30 minutes post-treatment (p=0.54). At 60 minutes, mean (SD) BP of the appropriate group was 177/97 (28/21) mmHg compared to 172/97 (26/16) mmHg in the inappropriate group (p=0.19). Hypotension developed in three patients, one of whom was being treated for suspected hypertensive emergency and two of whom had no documented suspicion for end-organ injury. These latter two patients required initiation of vasopressor support. One patient in each group developed iatrogenic bradycardia after use of labetalol that required the administration of atropine and additional telemetry monitoring. There was no statistical difference in in-hospital mortality between patients treated appropriately (2%) versus those treated inappropriately (0%). In addition, rates of 30-day ED revisit rates were high but equivalent (18.3% versus 17.3% respectively).

## DISCUSSION

Published data regarding management of severe hypertension with bolus IV antihypertensive therapy in the ED setting are limited. In this single center study, we found that more than one-third of patients with elevated BP who received bolus IV antihypertensive therapy in our ED received it inappropriately. Although we found only a few cases where this resulted in potential harm, this practice is contrary to current recommendations from ACEP to avoid BP reduction in asymptomatic patients in the ED.^7^ We suspect that in most cases of inappropriate treatment, rapid BP lowering occurs out of convenience. Anecdotedly, emergency physicians commonly describe that they rapidly improve a patient’s BP to “look better” upon discharge to home or transfer to an inpatient bed. Hospital clinicians may also request BP normalization prior to transfer to an inpatient bed.

Because acute management of chronically uncontrolled hypertension has not been shown to improve long-term outcomes and in fact could be detrimental, care is warranted in deciding which patients may benefit from IV antihypertensive bolus medications.^11^,^14^,^15^ Patients presenting with suspected or confirmed hypertensive emergency should continue to be managed with IV antihypertensive medications according to evidence-based recommendations. For patients presenting with severe BP elevation absent concern for hypertensive emergency, clinicians should be cognizant of the risks of IV antihypertensive therapy and manage these patients through appropriate oral antihypertensive regimens in conjunction with their primary care providers. While our study did not demonstrate a statistically significant difference in adverse effects or in-hospital mortality for patients in the group without documented suspicion for end-organ injury, two patients developed hypotension that required vasopressor treatment, suggesting the potential for serious consequences with indiscriminate use of IV antihypertensive therapy.

Although not directly assessed in our study, widespread use of IV antihypertensive therapy has other consequences including contributing to increased costs associated with IV treatment and critical drug shortages. Inappropriate use of labetalol, by far the most common medication given in our setting, in particular may be problematic as this drug has many indications for management of hypertensive emergency but has been in short supply at various points over the last five years.^16^ Automated order queries with indication-specific order justifications in the EMR could be implemented to reduce inappropriate use of IV antihypertensive therapy.

## LIMITATIONS

Our study has a number of limitations. Although we captured all available patients over the study time period by pharmacy records, the final number of patients was relatively small for the overall number of ED visits and limited to one site. Because of the nature of retrospective chart abstraction, the characterization of patients was dependent upon available documentation. Unknown factors may have contributed to treatment decisions that could not be accounted for with available documentation. Nevertheless, in the experience of the authors, treatment of severe hypertension with IV medications is commonly performed for the convenience of rapid lowering rather than clinical necessity. Also, while we found no evidence of harm with treatment of patients without documented suspicion for end-organ injury, more subtle adverse events such as confusion, mild stroke, or acute kidney injury related to hypoperfusion may have been underreported or unidentifiable through chart abstraction. We did not gather complete follow-up data from other health systems, limiting the assessment of readmissions and adverse events within 30 days. Lastly, the patients in this study were 93% African American and 88% had a known history of hypertension. The results of this study may not apply to different patient populations.

## CONCLUSION

In this cohort, IV antihypertensive therapy was administered inappropriately to patients without documented suspicion for end-organ injury nearly one third of the time. Systematic efforts to curtail this practice could have a lasting impact on healthcare resource utilization and warrant further exploration.

## Figures and Tables

**Figure:**
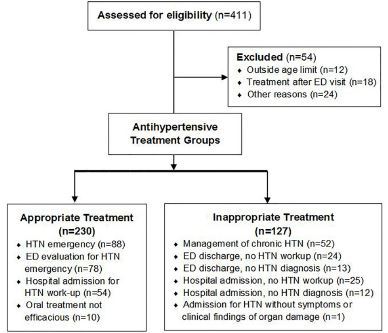
Flow diagram of participants* in a study that examined frequency of bolus intravenous antihypertensive treatment when hypertensive emergencies were not present. *ED*, emergency department; *HTN*, hypertension.

**Table 1:** Baseline demographics and characteristics of patients in a study examining the appropriateness of antihypertensive bolus administration when no true hypertensive emergency was present.

Characteristic	All patients (n = 357)	Appropriate use (n = 230)	Inappropriate Use (n = 127)	p-value
Demographics				
Age, years (mean ± SD)	54.7 ± 13.9	56.5 ± 13.8	51.4 ± 13.5	< 0.01
Male sex	183 (51.2)	117 (50.9)	66 (52)	0.84
African American	332 (93)	218 (94.8)	114 (89.8)	0.08
Past medical history				
Hypertension	315 (88.2)	210 (91.3)	105 (82.7)	0.02
Diabetes	91 (25.5)	63 (27.4)	28 (22)	0.27
Coronary artery disease	55 (15.4)	42 (18.3)	13 (10.2)	0.04
Chronic kidney disease	55 (15.4)	43 (18.7)	12 (9.5)	0.02
Heart failure	47 (13.2)	40 (17.4)	7 (5.5)	< 0.01
Stroke	31 (8.7)	23 (10)	8 (6.3)	0.23
No past medical history	31 (8.7)	15 (6.5)	16 (12.6)	0.05
Social history				
Tobacco use	141 (39.5)	89 (38.7)	52 (40.9)	0.68
Alcohol use	66 (18.5)	29 (12.6)	37 (29.1)	< 0.01
Cocaine use	31 (8.7)	23 (10)	8 (6.3)	0.23
Heroin use	21 (5.9)	13 (5.7)	8 (6.3)	0.80
Presenting symptoms				
Shortness of breath	86 (24.1)	76 (33)	10 (7.9)	< 0.01
Chest pain	64 (17.9)	51 (22.2)	13 (10.2)	< 0.01
Headache	47 (13.2)	30 (13)	17 (13.4)	< 0.01
Altered mental status	38 (10.6)	32 (13.9)	6 (4.7)	< 0.01
Numbness or weakness	33 (9.2)	31 (13.5)	2 (1.6)	< 0.01

* All values represented as n(%) unless otherwise indicated.

**Table 2:** Antihypertensive medication administration.

First dose of IV antihypertensive	All patients n = 357	Appropriate use n = 230	Inappropriate use n = 127	p-value
Labetalol	217 (60.8)	131 (57)	86 (67.7)	0.08
Enalaprilat	65 (18.2)	51 (22.1)	14 (11)	< 0.01
Hydralazine	64 (17.9)	39 (17)	25 (19.7)	0.59
Metoprolol	11 (3.1)	9 (3.9)	2 (1.6)	--
Second dose of IV antihypertensive (n = 86)				
Labetalol	57 (66.3)	43 (66.2)	14 (66.7)	0.87
Enalaprilat	17 (19.7)	4 (6.2)	2 (9.5)	0.62
Hydralazine	6 (7)	13 (20)	4 (19)	0.87
Metoprolol	6 (7)	5 (7.7)	1 (4.8)	--

*IV,* intravenous.
